# Could a Proto-Ribosome Emerge Spontaneously in the Prebiotic World?

**DOI:** 10.3390/molecules21121701

**Published:** 2016-12-09

**Authors:** Ilana C. Agmon

**Affiliations:** 1 Institute for Advanced Studies in Theoretical Chemistry, Schulich Faculty of Chemistry–Technion–Israel Institute of Technology, Haifa 32000, Israel; chilana@tx.technion.ac.il; Tel.: +972-4-829-3824; 2 Fritz Haber Research Center for Molecular Dynamics, Hebrew University of Jerusalem, Jerusalem 91904, Israel

**Keywords:** ribosome, proto-ribosome, ribosome symmetrical region, RNA world, origin of life

## Abstract

An indispensable prerequisite for establishing a scenario of life emerging by natural processes is the requirement that the first simple proto-molecules could have had a realistic probability of self-assembly from random molecular polymers in the prebiotic world. The vestige of the proto-ribosome, which is believed to be still embedded in the contemporary ribosome, is used to assess the feasibility of such spontaneous emergence. Three concentric structural elements of different magnitudes, having a dimeric nature derived from the symmetrical region of the ribosomal large subunit, were suggested to constitute the vestige of the proto-ribosome. It is assumed to have materialized spontaneously in the prebiotic world, catalyzing non-coded peptide bond formation and simple elongation. Probabilistic and energetic considerations are applied in order to evaluate the suitability of the three contenders for being the initial proto-ribosome. The analysis points to the simplest proto-ribosome, comprised of a dimer of tRNA-like molecules presently embedded in the core of the symmetrical region, as the only one having a realistic statistical likelihood of spontaneous emergence from random RNA chains. Hence it offers a feasible starting point for a continuous evolutionary path from the prebiotic matter, through natural processes, into the intricate modern translation system.

## 1. Introduction

The origin of life and the evolution of life are of widespread interest, but in spite of the efforts aimed at answering these questions, the current knowledge on the subject is limited and there are manifold hypotheses. An indispensable prerequisite for constructing a scenario for the emergence of life via natural processes is the ability to demonstrate a realistic probability for self-assembly of the first simple proto-molecules in the prebiotic world, prior to the existence of any replicative system. The challenge posed by this requirement is self-evident. Even the sequence of a simple ribozyme of 40 mer has 10^24^ possible compositions. To represent all of these compositions at least once, and thus to establish a certainty that this simple ribozyme could have materialized, requires 27 kg of RNA chains, which classifies spontaneous emergence as a highly implausible event [1].

Any scenario describing the emergence of “life as we know it”, i.e., biology based on nucleic-acid and amino-acid polymers, must include a proto-ribosome, which would have catalyzed the formation of a peptide bond between two amino acids and produced simple peptides. This proto-ribosome could have emerged and functioned within an RNA world [2,3,4], where RNA, perhaps with the aid of co-factors, acted as both the catalytic tool and the heredity source. Alternatively, it may have emerged spontaneously, as a stable molecule in the prebiotic chemistry. The ribosome, the contemporary apparatus responsible for catalyzing peptide bond formation during the translation of the genetic code into proteins, may offer a reliable source of extant information on its prebiotic ancestor, because of its universal nature, employing a common mode of action in all life domains. This, alongside with its considerable level of phylogenetic conservation, imply that the essence of the current translation mechanism should have already been present in the Last Universal Common Ancestor (LUCA) and that the vestige of the stand-alone primordial proto-ribosome may still be inferred from its contemporary structure. Owing to the accepted notion that the ribosome could not have emerged in its present complex form, it must have continuously evolved from a simple ancestor which took part in the emergence of the current mode of life. Its identification within the modern ribosome may therefore enable the examination of the feasibility of spontaneous materialization of a functional ribozyme in the prebiotic era.

**Figure 1:**
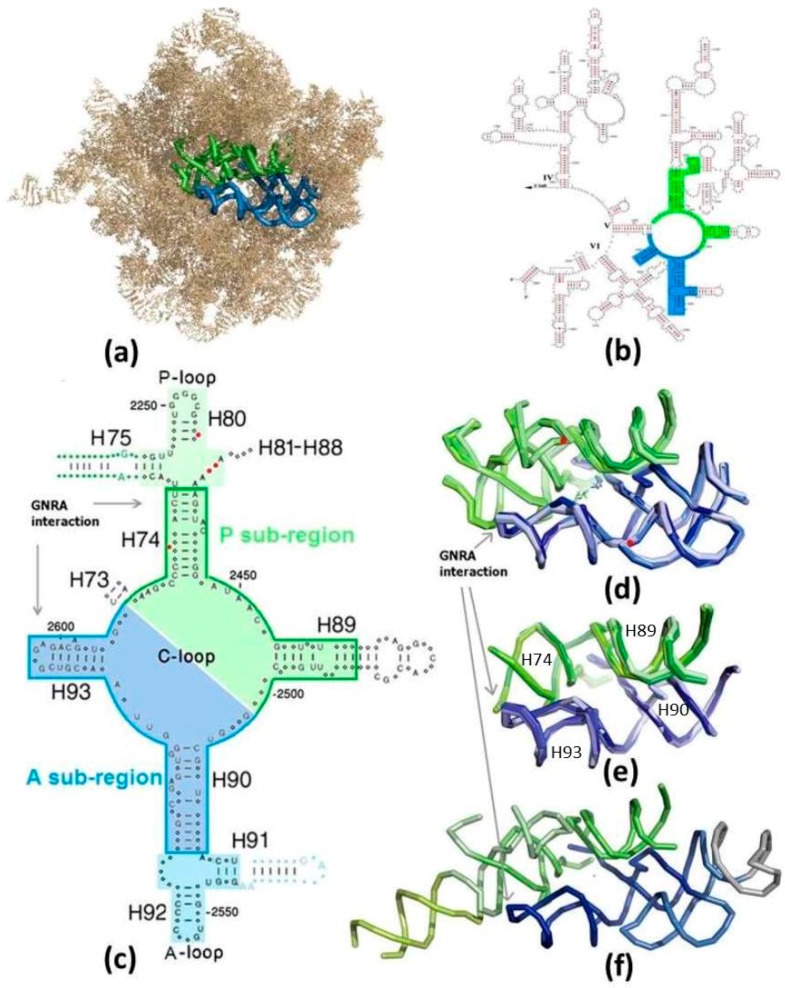
The suggested proto-ribosomes: (**a**) The symmetrical region (SymR) within the 23S ribosomal RNA (rRNA) of *E. coli* large subunit (pdb 2AW4). A-sub-region in blue, P-sub-region in green (color code maintained in all figures). H68-H71 were removed to expose the SymR; (**b**) The SymR within the secondary scheme of the 3’ half of 23S RNA from *E. coli* [25], drawn in a manner not portraying the symmetry; (**c**) 2D scheme of the zone encircling the PTC, drawn in a manner exhibiting the symmetry. Nucleotides conserved by more than 97% in each of the three domains of life are indicated by capital letters. Indels are marked by red dots. The core of the SymR, suggested to be the remnant of the dimeric proto-ribosome (DPR, boundary marked), together with the A-, P-sites (on lighter background), compose the SymR. The non-symmetrical parts of H75 and H91, indicated by blue and green letters and dots, extend from the SymR, together forming the ext-SymR; (**d**) Overlap of the SymR fold, with the PTC at its heart, projected along the symmetry axis, as found in the high resolution structures of archaea (pdb 1VQ6), three bacteria (T50S, pdb 2WDL; D50S, pdb 2ZJR; *E. coli*, pdb 2AW4) and eukarya (*S. cer*, pdb 3U5D), together with the reacting amino acid analogs (pdb 2WDL, 1VQ6). Nucleotides G2553 (*E. coli* numbering throughout the text) from the A-loop and G2251 from the P-loop, which symmetrically base-pair to C75 of the modern A-, P-tRNA substrates, are indicated by red dots. Peptide bond is formed at the bottom of the cavity; (**e**) The overlap of the DPR folds (same pdb files as in (**d**)); (**f**) The fold of the extended symmetrical region (ext-SymR, pdb 2AW4). The dimeric proto-ribosome is drawn in darker hue, the A-, P-sites in lighter hues and the non-symmetrical extensions of H75 and H91 in lime and grey, respectively.

In the contemporary ribosome, peptide bond formation, the essential function of the ribosome, takes place at the active site of the large subunit, i.e., at the Peptidyl Transferase Center (PTC), which is composed solely of RNA [5,6]. The PTC pocket harbors the universally conserved 3′ end of the Aminoacyl-tRNA substrate, carrying the incoming amino acid and the 3′ end of the Peptidyl-tRNA, bearing the nascent chain synthesized up to that point. The PTC is located at the heart of a region of about 180 nucleotides, possessing an approximate 2-fold rotational symmetry [7,8]. The two symmetry-related sub-regions of the symmetrical region (SymR) are termed the A-, P- sub-regions (Figure 1), after the A-, P-sites contained in them. The structure of the SymR (Figure 1a–d) is conserved throughout all of the high resolution structures of the large subunit determined so far, from the three domains of life (Figure 1d), i.e., in the structures of archaea—*Haloarcula marismortui* (H50S) [9]; bacteria—*Deinoccocus radiodurans* (D50S) [10], *E. coli* [11], *Thermus thermophilus* (T50S) [12]; eukarya—*Saccharomyces cerevisiae* (*S. cer*) [13], as well as in mitochondrial ribosomes [14,15]. In addition to its 3D structural preservation, this region is highly conserved phylogenetically [8]. Thus, a version of this symmetrical RNA element, which conforms to the exact stereochemistry of the current PTC, may be conceived as a stand-alone proto-ribosome. Taking advantage of the RNA tendency to form stable dimers spontaneously [16,17,18], such an RNA element can be described as a dimer, whose spontaneous materialization in the prebiotic world would have depended on the aptitude of its monomers to self-assemble correctly from random RNA chains.

The inverse ratio between the length of the ribozyme sequence and the probability of its autonomous formation implies that a dimeric nature of an enzyme increases the feasibility of its random emergence by many orders of magnitude. Three concentric proto-ribosomes of dimeric nature were suggested: The entire SymR of about 180 nucleotides (Figure 1c,d), whose hub hosts peptide bond formation in the modern ribosome, was linked with the proto-ribosome due to its role, its universality and the high conservation of its structure [19]. A unique pattern of A-minor interactions [20] found within the ribosome, constantly pointing from the periphery of the rRNA into the SymR, was interpreted as presenting a mode for adding new structural elements to an existing proto-ribosome [21]. This observation yielded a proto-ribosome model of 225 nucleotides, composed of the SymR extended by the non-symmetrical parts of H75 and H91 (Figure 1c,f), denominated here the extended symmetrical region (ext-SymR). A smaller element of about 120 nucleotides, termed the dimeric proto-ribosome (DPR), constituting the core of the SymR (Figure 1c,e), was concurrently suggested to be the initial proto-ribosome [22,23,24]. Eliminating the vicinity of the A-, P-loops from the SymR structure leaves a dimer of two L-shaped RNA elements, the A-, P-DPR monomers, which are comparable in size and shape to the tRNA molecules [23,24]. These three structural entities preserve the stereochemistry of the amino acids’ accommodation at the A- and P-sites of the modern ribosome and could thus have potentially acted as catalysts for the prebiotic peptide bond formation, prior to the advent of mRNA and coding.

The probability that any of the monomers of the three suggested proto-ribosomes could have spontaneously emerged as a building block of the first ribozyme catalyzing non-coded peptide bond formation is hereby assessed. By introducing the notion of "limited specificity” in this context, the feasibility of a spontaneous materialization of the simplest contender, the dimeric proto-ribosome, is demonstrated.

## 2. Results

The zone encircling the PTC, from where the three proto-ribosome models were derived, exhibits essentially full conservation of the secondary and tertiary structures across the three domains of life (Figure 1c,d). The structural preservation is manifested in the low RMSD between the X-ray structures from various ribosomes; an RMSD < 1.1 Å between pairs of atomic structures of the bacterial SymR and ≈1.4 Å between the eukaryotic SymR and the bacterial ones. This structural preservation, along with the high sequence conservation across the three life domains [8], implies that the three contenders resemble their prebiotic ancestors. The contemporary sequences of this region may thus allow the assessment of the probability that a proto-ribosome could have spontaneously emerged in the prebiotic world.

### 2.1. The Probability of Random Occurrence of a Proper Proto-Ribosome Sequence

The probability of a random occurrence of a proper sequence, that could have comprised the initial proto-ribosome, was computed for the three concentric models of dimeric nature (Figure 1c–f): the entire symmetrical region (SymR, 178 nucleotides in bacteria, nucleotides included: P-SymR monomer: 2058–2079; 2241–2258; 2430–2463; 2487–2501; A-SymR monomer: 2502–2522; 2543–2610), its core (DPR, 121 nucleotides in the three domains of life, nucleotides included: P-DPR monomer: 2058–2074; 2435–2463; 2487–2501; A-DPR monomer: 2502–2517; 2567–2610) and the extended symmetrical region (ext-SymR, 225 nucleotides in bacteria, nucleotides included: P-ext-SymR monomer: 2058–2092; 2227–2258; 2430–2463; 2487–2501; A-ext-SymR monomer: 2502–2610).

The three proposed proto-ribosomes share a common dimerization mode (Figure 1c–f) via GNRA interaction motif [26], in which a terminal GNRA stem loop (N = any nucleotide; R = purine) recognizes a helical receptor region. In congruence with the general tendency of RNA to form stable dimers [16,17,18], the GNRA dimerization mode was shown to contribute significantly to the stability of corresponding RNA dimers [27,28]. Moreover, the stabilizing effect gained by the dimerization of the SymR and the DPR monomers was demonstrated by quantum mechanics computation [29]. The three proto-ribosome contenders could have therefore self-assembled, in an energetically downhill process, providing that their monomers had had suitable sequences that could have folded correctly, preserving the structure and the relevant functional components found in the PTC of the contemporary ribosome. 

This section attempts to identify the minimal set of constrains on the nucleotide's type which would have allowed a monomer of each of the three contenders to fold into a working proto-ribosome, and to estimate the probability of random occurrence of such a hypothetical oligonucleotide. The constraints are derived under the requirement that any hypothetical sequence should ensure the preservation of the present ribosomal mechanism of peptide bond formation. Hence, in order to acquire the conserved spatial organization of the PTC, the hypothetical 2D scheme should conform to the secondary structure found within the contemporary ribosome. Additionally, the identity of a subset of essential nucleotides involved in indispensable roles should be retained.

Two types of essential nucleotides would have been present in the initial proto-ribosome: those involved in accommodating the substrates and those responsible for stabilizing the tertiary structure. The peptide bond reaction mechanism is common to the three domains of life, as indicated by the X-ray structures of their ribosomes. It is therefore reasonable that the same basic mechanism would have been utilized by the proto-ribosome, implying that the proto-ribosome accommodated the reacting amino acids in the same manner as the modern ribosome does, i.e., against the walls of the PTC (Figure 1d). The PTC walls are primarily composed of single stranded RNA segments from the central loop of domain V (C-loop), and this pocket is stabilized by the four helices—H74, H89, H90 and H93 radiating from it (Figure 1c,e). It follows that those C-loop nucleotides, which are fully conserved in the three domains of life, are probably functionally indispensable for the modern mechanism and would retain their nucleotide type in any hypothetical sequence suggested here for the proto-ribosome.

The larger optional proto-ribosomes, namely, the SymR and ext-SymR, contain the A-, P-loops (Figure 1c,d,f) which, in the PTC of the modern ribosomes, base-pair to the 3’ end of the aminoacyl- and the peptidyl-tRNAs. Accordingly, the SymR and ext-SymR proto-ribosomes are assumed to have used the A-, P-loops for hosting their substrates. Hence, in addition to the conserved C-loop nucleotides, the fully conserved A- and P-loop nucleotides should also keep their present type in any hypothetical sequence suggested for the larger proto-ribosomes.

Additionally, several nucleotides involved in stabilizing the 3D structure should hold a particular nucleotide type. In order to maintain the exact dimerization mode which determines the outline of the PTC [23,24], the nucleotides involved in the dimer forming GNRA interaction motif (Figure 1c–f) are required to hold the modern nucleotide type. Conversely, variations in the stem’s sequence, such as exchanging one base-pair with another, are tolerable as long as the secondary structure is maintained. Indeed, in the contemporary ribosome, the C-loop, A-loop, P-loop and the GNRA motif nucleotides are highly conserved (Figure 1c), while the stem nucleotides, which constitute the majority of the suggested proto-ribosomes sequences, show considerable base-pair variation among species.

Nucleotides are therefore considered here to be essential for the function of the proto-ribosome if they reside on the C-loop, A-loop, P-loop or are dimer forming nucleotides, having conservation level >97% in each of the three domains of life (see Materials and Methods). Assuming an even distribution of the four nucleotides, the probability of randomly obtaining a specific nucleotide type is 25%. Essential nucleotides, which are restricted to a specific identity, were therefore assigned a probability of 0.25 to be randomly found with the desired nucleotide type. Base-paired nucleotides, required to retain the base-pairing while allowing for variations in their type, were assigned a 0.5 probability to be randomly obtained with the correct nucleotide identity. The less conserved loop nucleotides, as well as the bulged stem nucleotides, were permitted to have any type, and were thus assigned a probability of 1.0 for being randomly obtained with the correct nucleotide type. Several bulged nucleotides, despite their full conservation in the contemporary ribosomes, were still assigned a random nucleotide type in the hypothetical sequences, because the current conservation is likely to be associated with their roles in functions which evolved subsequently. A2577 and C2573 of H90, for example, are 100% conserved in all the modern ribosomes, the former probably because it stabilizes the SymR against the ribosome bulk and the latter most likely due to its role in translocation [8], functions which are both inapplicable to this initial proto-ribosome.

**Figure 2:**
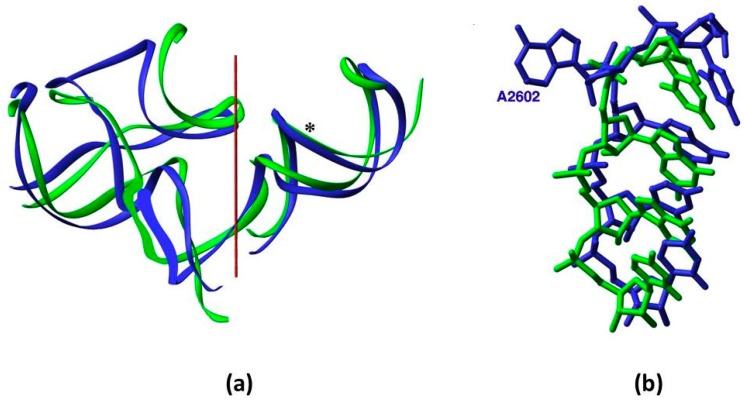
Symmetry: (**a**) Overlap of the backbone fold of the A- and the P-sub-regions, obtained by rotating the A-sub-region by 179.6° around the symmetry axis (in red); (**b**) The compatibility between the nucleotide conformation in the overlapped segments of the symmetry-related H89 and H93, whose backbone is marked by an asterisk in (**a**). Nucleotide A2602, which was suggested to be actively involved in translocation, diverges from the symmetry and bulges into the essentially symmetric PTC [7,8].

Due to the symmetry which relates the two monomers (Figure 2) of each contender (except for a limited non-symmetrical periphery in ext-SymR (Figure 1c,f)), the probability of the random occurrence of a functional sequence was examined by comparing a single monomer of each of the three proto-ribosomes. The analysis was carried out on the A-monomers, whose secondary structure is completely conserved in the three domains of life. The sequences of the P-monomers vary slightly across the phylogenetic domains (Figure 1c), having few inserted or deleted nucleotides (indels).

#### 2.1.1. Probability of Random Occurrence—the DPR Monomer Sequence

The vestige of the A-monomer of the dimeric proto-ribosome holds 60 nucleotides (Figure 1c). If complete conservation of the sequence found within the ribosome would have been required, the probability *P* of randomly obtaining this predetermined sequence would be given by:

*P* = 0.25^60^ ≈ 10^−36^ (1)

The amount of random chains required in order to get at least one oligonucleotide with this specific sequence, with a probability larger than 99% (see Materials and Methods), will require at least 10^36^ oligonucleotides of about 60 mer. Consequently, one liter of 1 mM solution is expected to contain 10^−15^ RNA chains with the desired sequence, or, in other words, a volume of 10^15^ L, which is equivalent to 1000 km^3^ of the solution, is needed in order to find at least one chain with the desired sequence.

When, however, only partial sequence conservation is required, which means that only a fraction of the nucleotides in the sequence are required to hold a specific type, the odds improve dramatically. The conservation requirements for the A-DPR monomer demand that the eight C-loop nucleotides which are fully conserved in the three domains of life (Figure 1c) should maintain their nucleotide identity in any hypothetical sequence suggested for the proto-ribosome. In addition, if dimerization through GNRA interactions is assumed to occur on both sides of the proto-ribosome pocket [23,24], the nucleotides involved in that motif, i.e., the 100% conserved H93 loop nucleotides G2595 and A2598 and the receptor base-pairs C2517:G2567 and A2516:U2568 (symmetry-related to the 100% conserved U2074:A2435 and C2073:G2436 of H74) should maintain the nucleotide identity found in the suggested vestiges of the proto-ribosome. These 14 essential nucleotides are therefore assigned a probability of random occurrence of 0.25. The nucleotides involved in the 15 additional base-pairs in the A-DPR monomer, which have to be retained (allowing the base-pair type exchange) and the “R” nucleotide from the GNRA loop which has to be a purine, all have a 0.5 probability of being randomly obtained with the correct type. The six less conserved nucleotides in the C-loop, as well as the eight non-base-paired helix nucleotides and the “N” nucleotide in the GNRA loop of H93 (Figure 1c) are all permitted to have a random type. Under these relaxed requirements for nucleotide type specificity, the probability of obtaining a functional version of the DPR monomer, which can potentially assemble a ‘working’ dimeric proto-ribosome, is increased to:

*P* = 0.25^14^ × 0.5^31^ × 1^15^ ≈ 10^−18^ (2)

Such a probability of formation indicates that each liter of 1mM solution of random RNA chains of about 60 mer would have included more than 500 A-DPR-like sequences, predisposed to form L-shaped DPR monomers with dimerization affinity and conserved reactant accommodation position.

It is also possible that the initial dimerization mode was simpler than the current one [23,24], occurring via an A-minor interaction motif [20] which makes part of the full GNRA motif. In this case only A2598 and the A2516:U2568 base-pair from among the dimer-forming nucleotides would be considered as essential and the probability of occurrence of an A-DPR-like sequence would further increase to:

*P* = 0.25^11^ × 0.5^32^ × 1^17^ ≈ 5 × 10^−17^ (3)

#### 2.1.2. Probability of Random Occurrence—the SymR Monomer Sequence

Identical considerations to those applied to the A-DPR monomer are utilized for the larger A-SymR monomer (Figure 1c). Adding the three completely conserved nucleotides U2552, G2553 and C2556 from the A-loop, that accommodate the 3’ end of the modern substrates, with probability 0.25 of random occurrence, the 14 base-paired nucleotides from H91, H92 each with probability 0.5 per nucleotide, and the remaining twelve nucleotides with a random type, gives:

*P* = 10^−18^ × 0.25^3^ × 0.5^14^ × 1^12^ ≈ 10^−24^ (4)

representing a significantly lower probability of random occurrence of an appropriate 89 mer SymR monomer, compared to the DPR monomer.

#### 2.1.3. Probability of Random Occurrence—the Ext-SymR Monomer Sequence

The A-ext-SymR monomer is composed of the A-SymR monomer appended with the non-symmetrical extension of H91, which includes four fully conserved non-base-paired nucleotides (Figure 1c). These nucleotides are, however, too remote from the PTC to be involved in the indispensable roles defined above, and are therefore assigned a probability of 1 to be randomly found with the correct type. Incorporating the twelve base-paired nucleotides of the H91 non-symmetrical extension with probability 0.5 of random occurrence per nucleotide, to the probability calculated for the A-SymR, gives the following probability for the A-ext-SymR monomer of 109 mer:

*P* = 10^−24^ × 0.5^12^ × 1^8^ ≈ 10^−28^ (5)

representing the lowest probability of random occurrence of an appropriate sequence among the three suggested proto-ribosomes.

### 2.2. Fold Predictions of Ribosomal Sequences 

The contemporary sequences of the three contenders, obtained from the ribosomes whose structure was determined by X-ray, are examples of sequences that carry the full set of constraints on the nucleotide identity defined in Section 2.1.1, Section 2.1.2 and Section 2.1.3. As such, they can be used to test the capability of a proper hypothetical oligonucleotide to self-fold into the secondary structure found within the modern ribosomes. The folds of the monomeric sequences were predicted (Table 1), by applying free energy minimization using the program Mfold [30] (see Materials and Methods).

**Figure 3:**
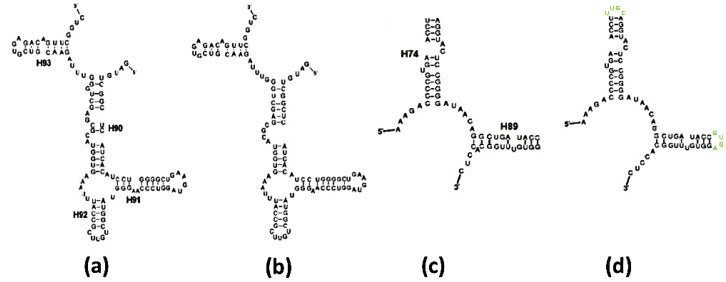
Ribosomal vs. predicted secondary schemes: (**a**) Scheme of the A-ext-SymR monomer from *E. coli*; (**b**) Mfold prediction of the sequence in (**a**); (**c**) Scheme of the P-DPR monomer from *E. coli*; (**d**) Mfold prediction of the sequence in (**c**), with stem loops artificially appended to the truncated H74, H89 (in lime) to obtain an intact chain.

The six predictions obtained for the eukaryotic sequences of the A-, P-monomers from the three suggested proto-ribosomes didn't match the secondary structures found in the modern ribosomes. Application of free energy minimization to the ≈90 mer sequences of the prokaryotic A- and P-monomers of the SymR, appended with tetra loops to complete the truncated helices H75, H89 and H91 (Figure 1c), led in all cases but one to several optional folds, hardly differing in their free energy. Just two of the best solutions had a secondary structure resembling that found within the contemporary ribosome. The best prediction-results for the ≈110 mer sequences from the A-, P-monomers of the prokaryotic ext-SymR (Figure 1c and Figure 3a,b) were mostly compatible with the secondary scheme found within the ribosome, but only some of them were unique. In contrast, the 60 mer sequences from the prokaryotic DPR monomers (Figure 1c and Figure 3c), appended with completing stem-loops, led, in all cases but one, to a unique solution. The sequences were consistently predicted to fold into L-shaped RNA elements, compatible with their structure in the ribosome (Figure 3c,d). However, slight deviations from the exact base-pairing scheme were found even in fold predictions of sequences considered to be correctly folded (Figure 3a–d).

For the correctly folded sequences, the free energy of formation lie in the range ΔG= −25 ± 3 kcal/mole for the prokaryotic DPR monomers, and for the larger contenders, i.e., the SymR and the ext-SymR monomers, having additional base-pairs, in the range ΔG= −32 ± 4 kcal/mole and −45 ± 4 kcal/mole, respectively.

#### Folding of Randomized Sequences 

**Figure 4:**
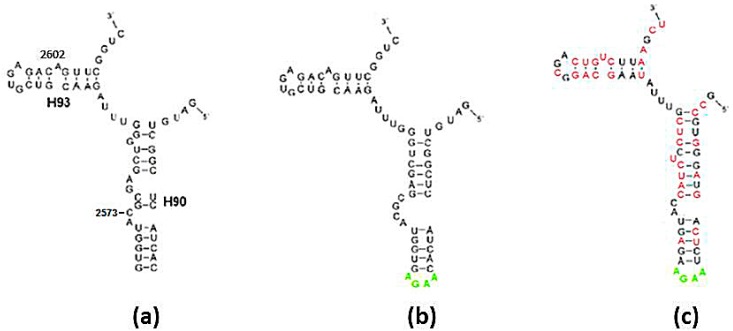
Folding of the A-DPR sequence: (**a**) The secondary structure found within the contemporary ribosome; (**b**) Fold prediction for the sequence in (**a**); (**c**) Fold prediction for the sequence in (**a**,**b**), after randomly mutating over 50% of the nucleotides, subject to the constraints determined in Section 2.1.1. Nucleotides with exchanged types are drawn in red. The stem loop of H90 (in lime) in (**b**,**c**) were artificially added to obtain an intact chain.

As an additional benchmark, the folds of ten randomized sequences of the 60 mer A-DPR monomer from *E. coli* were predicted (Figure 4a–c). Over 50% of the nucleotides were mutated, while the 14 essential nucleotides and the 15 base-pairs were assigned the suitable nucleotide type according to the restrictions derived in Section 2.1.1. All ten randomized sequences folded into L-shaped RNA elements, in agreement with the A-DPR secondary structure (Figure 1c and Figure 4a), but only half of them acquired folds analogous to the 2D scheme found in the ribosome (see Materials and Methods for the definition of “analogous” in this context). Local deviations in the predictions of the randomized sequences occurred in the same region of H90 that deviated in the folding-predictions of the modern sequences (Figure 3a,b and Figure 4a–c). Additionally, the predictions of the randomized sequences always exhibited extra base-pairs formed between nucleotides belonging to the single stranded C-loop (Figure 4c). 

## 3. Discussion

The preservation of the symmetrical region structure in ribosomes from bacteria, archaea and eukarya (Figure 1c,d), suggests that this fraction of the ribosome carries structural features older than the differentiation into the life kingdoms. The ancestor of the SymR would have therefore been part of LUCA. As such it would have already been capable of translating a code in a processive manner, implying that beyond applying positional catalysis to the substrates, this molecular machine would have been adapted to some properties of the modern mechanism, such as translocation and substrates conjugated with anticodon loops. This apparatus, whose vestige is suggested to build the modern PTC in the large subunit, should have therefore been an evolved version of the earliest non-coded proto-ribosome, i.e., a proto-ribosome that did not use a code. The primal entity is assumed to have emerged spontaneously as a stable molecule in an era of “chemical evolution”, an epoch which initiated and proceeded in the absence of polymerases, heredity, genetic information, and Darwinian evolution [31]. This non-coded proto-ribosome would have catalyzed peptide bond formation between random amino acids by exerting positional catalysis on small substrates and enabled simple elongation [19,22,23,24].

The feasibility of self-assembly of a ribozyme from prebiotic random RNA chains is a question central to the ability to conceive life emerging by natural processes. In the quest for retracing the initial autonomously-formed proto-ribosome, the ensuing coded version conserved in the current ribosomes is used as a model for the non-coded proto-ribosome. The usage of the advanced version is justified because the DPR, representing a vestige of the coded proto-ribosome, was demonstrated to be the smallest possible apparatus of its kind that can preserve the PTC layout [23,24]. If the non-coded proto-ribosome could not have been smaller than this coded one, and if, as assumed, it underwent a continuous evolution towards the proto-ribosome present in LUCA, it is reasonable that the initial and evolved versions were similar. Subsequent modifications, which are present in the coded proto-ribosome due to its adaptation to the more complex function, present a challenge to the derivation of the constraints on the nucleotide identity required for obtaining a proper hypothetical sequence of the non-coded proto-ribosome. Additionally, these modifications, combined with the limited information available concerning the nature of the early substrates and the environmental conditions that prevailed on the prebiotic earth, probably impede the attempts to generate a working non-coded proto-ribosome [32]. Further clarification of these uncertainties may enable the production of a functioning proto-ribosome in the lab.

Three suggested vestiges of the coded proto-ribosome, having a dimeric nature, were derived from the structure of the modern ribosome; the entire symmetrical region—i.e., the SymR [19], its core—the DPR [22,23,24] and the SymR extended with the non-symmetrical parts of H75 and H91 [21], namely, the ext-SymR (Figure 1c–f). The shortest oligomer is required for the formation of the A-DPR monomer—about 60 mer, while the longest is that of the P-ext-SymR monomer, 116 mer. Random RNA polymers of over 100 mer are believed to have existed in the prebiotic environment based on the polymerization of nucleotides on clay [33], on the elongation of oligonucleotides under temperature gradient [34] and on the possibility of self-ligation of RNA chains [35], pointing to the prebiotic existence of oligonucleotides sufficiently long to form each of the monomers. 

The probability of random occurrence of a sequence capable of forming a structurally and functionally suitable monomer was tested under the notion of “limited sequence specificity” [23]. Limited specificity means that just a subset of residues, mainly those involved in functional or structural tasks, have to be restricted to a particular type in order to obtain a working enzyme. This concept is manifested by the A- and the P-sub-regions of the SymR, which in the modern ribosome primarily perform an equivalent role of symmetrically accommodating the reactants of peptide bond formation [6]. In accordance, the sub-regions exhibit significant 2D and 3D resemblance (Figure 1c and Figure 2), while their sequences are hardly related. In *E. coli* for instance, only 38% of the symmetry-related nucleotides hold an identical nucleotide type. Nevertheless, the functionally vital nucleotides G2553 from the A-loop and G2251 from the P-loop, which symmetrically accommodate the substrates by forming base-pairs to C75 of the modern A- and P-tRNAs, maintain the same nucleotide type in the two sub-regions. This supports the notion that ribozymes with varying sequences may execute equivalent roles, provided that the functional and structure-determining residues retain the required nucleotide type. Future experimental verification of this notion seems feasible. Mutating the sequence of a well-studied ribozyme, while preserving its secondary structure and the identity of its essential nucleotides, will permit the examination of the limited specificity effect on the function and perhaps result in more efficient ribozymes.

Limiting the requirement for specific nucleotide identity increases the probability of a random occurrence of a functional sequence by many orders of magnitude, compared with that of a fixed-sequence ribozyme of the same size, as demonstrated for the A-DPR monomer, i.e., 10^−18^ vs. 10^−36^, respectively. This concept may be applied to other specialized enzymes as well. A ≈ 190 mer ribozyme replicase created via directed evolution, with the ability to replicate a 95-nucleotide stretch of RNA [36], was considered to be far too long a sequence to have arisen through any conceivable process of random assembly [37]. If, however, the type of the non-catalytic and non-structural residues is permitted to vary, appropriate sequences other than the one obtained in the lab may exist, thus increasing the probability of random occurrence considerably. Additionally, the limited specificity is evolutionary advantageous, since it would have granted the prebiotic apparatus a tolerance to the rather poor copying abilities that any initial replicase would have probably had.

The probability of random occurrence of a proper sequence of the A-DPR-like monomer, i.e., ≈10^−18^, which pertains to the A- as well as to the P-DPR monomers, is probably naïve. A random mutation in a loop or bulged nucleotide, permitted here to have a random type, may induce alteration in the base-pairing scheme, decreasing the calculated probability. Still, among the nine A- and P-DPR sequences derived from the current ribosomes (Table 1) and the ten extensively randomized A-DPR sequences from *E. coli*, all of which comply with the full set of constraints on the nucleotides identity defined in Section 2.1.1, more than half were found to fold into structures analogous to those found within the ribosome (Figure 3 and Figure 4). This suggests a reduction of the probability assessed for random occurrence of the non-coded proto-ribosome sequence to 5 × 10^−19^.

Among the secondary structure predictions of the 27 contemporary sequences, performed using energy minimization with Mfold (Table 1), the eukaryotic sequences of the monomers of all three contenders were predicted to acquire folds different from those found within the ribosome. This result implies that the eukaryotic sequences are not directly related to that of the proto-ribosome and corroborates the notion that eukaryotes are a not direct descendants of LUCA [38,39]. The sequences of the prokaryotic SymR monomers were predicted to have multiple alternatives of equally probable folds, mostly incompatible with the suggested proto-ribosome structure. The odds of obtaining an RNA element having the SymR monomer fold from its contemporary sequences are therefore slight. In the case of the prokaryotic ext-SymR monomeric sequences, the most probable fold predictions are compatible with those found in the ribosome only for the bacterial sequences. Moreover, some of the predicted folds are non-unique and the multiple predictions hardly differ in their free energy, thus reducing the chance of spontaneously obtaining the correct secondary structure. 

Conversely, if any of the sequences of the DPR monomers found within the contemporary prokaryotic ribosomes existed in the prebiotic world, under environmental conditions which do not differ considerably from the default parameters used by the Mfold program, they would have been compelled to fold into an L-shaped structure matching the one found within the ribosome, in accord with the unique solutions obtained via energy minimization (Table 1, Figure 3c,d and Figure 4a,b). Moreover, half of the extensively randomized sequences of A-DPR from *E. coli* were predicted to acquire similar secondary schemes (Figure 4c). Consequently, sequences of about 60 mer, which conform to the set of constraints on the nucleotides’ identity defined in Section 2.1.1, can be considered as having a tendency to spontaneously fold into a secondary structure comparable to that found within the ribosome.

The probabilistic criterion, which is equally applied to the three suggested proto-ribosomes, considerably favors the DPR to the larger ones, due to its realistic probability of random occurrence, which is higher by six and ten orders of magnitude relative to the SymR and ext-SymR. Consistently, the energetic criterion points to DPR monomers as having more enhanced propensity to fold correctly. The compatibility of the DPR with the characteristics of the vestige of the non-coded proto-ribosome correlates well with its being structurally the simplest, as is to be expected from a prebiotic apparatus. Additionally, its 3D fold is completely conserved in the modern ribosome (Figure 1c,e), as opposed to the slight deviations found in a peripheral element of the SymR and the ext-SymR in different life domains (Figure 1d upper left), caused by the indels (Figure 1c). These arguments and the fact that the DPR building blocks i.e., its two L-shaped monomers, are comparable in size and outline to the L-shaped tRNA [22,23,24] considered to be a molecular fossil [40], point to the DPR being the most probable vestige of the proto-ribosome. 

The tendency of sequences of the prokaryotic A-DPR to fold correctly (Table 1) and to dimerize [27,28], suggests that the information embedded in its contemporary sequences may suffice for identifying the determinants for self-assembly into the desired structure. This, however, is not straight forward because the model derived from the modern ribosomes belongs to a more evolved proto-ribosome than the non-coded one. Local deviations between the ribosomal 2D scheme and the fold prediction of the same sequences are inevitable even in the sequences considered as correctly-folded, e.g., Figure 3a–d and Figure 4a,b, because the coded ribosome was adapted to advanced functions at the expense of losing stability. The contemporary structure of H90, for instance, contains a complex network of non-base-paired nucleotides, which is obviously less stable than the predicted one (Figure 3a,b and Figure 4a,b). Its spatial organization presumably enables C2573, a 100% conserved nucleotide from the non-base-paired region of H90 (Figure 4a), to bulge out into the PTC and carry out a role in translocation which is specific to coded proto-ribosomes [7,8]. The minimal free-energy structure predicted by Mfold represents the structure of an autonomously folded molecule. Hence, the similarity between the folding predictions and the secondary structure within the ribosome (Figure 3c,d and Figure 4a,b), imply that these folds are likely to constitute good approximations of the secondary structure of the non-coded proto-ribosome monomer. These folding results were obtained from sequences that comply with the restrictions on the nucleotide type determined in Section 2.1.1, thus supporting the constraints applied to the helical portion of the structure.

Another group of constraints pertain to the C-loop nucleotides. These nucleotides build the walls of the active site, and thus include the essential nucleotides involved in accommodating the reactants. The continuity principle would require that the exact mechanism of peptide bond formation is maintained, i.e., that the stereochemistry of the reactants in the active site of the non-coded proto-ribosome and of the modern ribosome must to be identical. In the modern ribosome the reacting amino acids rest upon nucleotides from the C-loop, but the actual accommodation takes place via base-pairing of the tRNA 3’ end with the A- and P-loops. The compact non-coded proto-ribosome that lacked the A, P-loops would have accommodated smaller substrates, whose atomic interactions with the C-loop nucleotides could have been different. The nature of these initial substrates is currently unknown and several options, such as amino-nucleotide conjugates similar to puromycin, or oligo-linked amino acids [41], as well as single amino acids [42], were already suggested. The more primitive mechanism implies that only part of the eight C-loop nucleotides essential for the modern function (Figure 1c), were vital for the non-coded mechanism, and that fewer C-loop nucleotides should be restricted to a particular type, thus increasing the overall calculated probability. On the other hand, extra base-pairs were formed between C-loop nucleotides in all the correctly-folded randomized sequences, a change that would have altered the layout of the PTC completely. To preserve the single stranded nature of the C-loop, additional or different constraints should be applied to the 14 C-loop nucleotides, possibly decreasing the overall calculated probability. The determined probability of the random occurrence of a sequence of the A-DPR-like monomer, 5 × 10^−19^, indicates that each liter of 1mM solution of random RNA chains of about 60 mer would have included about 300 oligonucleotides having sequences predisposed to form L-shaped DPR monomers with dimerization affinity and conserved reactant accommodation position. Thus, even a probability lower by two orders of magnitude would still suffice to grant the non-coded proto-ribosome an acceptable probability of self-assembly from random RNA chains. 

The vestige of the compact DPR within the current ribosome is comprised of 121 nucleotides, but as a stand-alone entity it would have had loops capping the currently truncated H74, H89 and H90 (Figure 1c). If capping by tetra loops is assumed, in accord with the existing H93 stem loop, the overall number of nucleotides in the DPR would have been 133. Hence its autonomous formation would have required two RNA chains of about 70 mer, a length comfortably within the range believed to exist in the prebiotic world [33,34,35]. A tiny fraction of these chains, i.e., those conforming to the constraints on the nucleotide type, would have spontaneously folded into the more stable L-shaped RNA molecules. Two monomers could then spontaneously dimerize around the 2-fold symmetry axis, via GNRA interactions, to form a pocket-like RNA entity (Figure 1e). Both folding and dimerization are energetically downhill processes, indicating that molecules of the dimeric proto-ribosome could have been readily available in the prebiotic world, catalyzing peptide bond formation at the bottom of the cavity in the same manner that the contemporary ribosome exerts positional catalysis; by accommodating the two reactants in a stereochemistry favorable for peptide bond formation. An efficient apparatus of this kind could have synthesized short peptides with random composition [22,23,24], which would have granted the proto-ribosome an evolutionary advantage of added stabilization, in analogy to the stabilization conferred to the contemporary rRNA by the ribosomal protein tails, which are believed to have a primordial origin [43,44,45].

The entire SymR, or more likely—the ext-SymR, was probably an evolved stage of the dimeric proto-ribosome with the additional RNA helices emanating from the far ends of H74 and H90 (Figure 1c). It is conceivable that the presence of random peptides, whose formation was catalyzed by the DPR, could have assisted the correct folding and stabilization of these more complex proto-ribosomes and possibly promoted the utilization of larger substrates. Some form of primitive replication would have emerged, either non-enzymatic or by very simple replicators, causing variability via mutations in the copied sequences and allowing for the selection of the fittest proto-ribosome, thus promoting evolution. Following the advent of coding, strings of RNA functioning as mRNA, would be translated by the coded proto-ribosome into the first coded peptides. Additional RNA elements would have later joined the proto-ribosome via different mechanisms [21,46] and this, along with the gradual incorporation of proteins serving in stabilizing and supporting roles, could have eventually led to the current complex ribosome.

The most fundamental requirement from a proper initial proto-ribosome model is its capability to autonomously emerge in the prebiotic world with the aptitude to catalyze peptide bond formation. The probability of spontaneous occurrence of the DPR sequence, obtained under the notion of limited sequence specificity, may be too optimistic, but even a significantly lower probability is still acceptable, taking into consideration that the emergence of life is believed to have occurred just once. Given optimal, yet unknown environmental conditions and sufficient time, a small pond with a relatively low concentration of random RNA chains of about 70 mer may have provided feasible likelihood for the materialization of a prebiotic apparatus catalyzing non-coded peptide-bond formation and simple elongation. This dimeric proto-ribosome thus offers a conceivable prebiotic starting point for a natural pathway leading to the complex protein biosynthesis mechanism, shared by all the modern living organisms.

## 4. Materials and Methods 

### 4.1. Nucleotide Conservation Level

The nucleotides having conservation level higher than 97% in each of the three life domains were detected in the CRW site [25]. Nucleotides whose percentage of conservation is around 97% were additionally examined against the sequences stored in the SILVA ribosomal RNA database [47]. 

### 4.2. Secondary Structure Predictions

The secondary structure of an RNA oligonucleotide was predicted by applying free energy minimization using the program Mfold [30] version 3.5, with default parameters, folding temperature fixed at 37 °C and window = 0.5. The program screens the fold-landscape of an input sequence and produces a list of thermodynamically favorable secondary structures, along with their free energy of formation.

Predicted folds were considered to be analogous to the ribosomal 2D scheme if they fulfilled three terms:

1. All the helices that appear in the ribosomal 2D scheme existed also in the predicted fold.
2. The stem loops were built from the same nucleotides in the ribosomal and the predicted folds.
3. Specifically, for the A-DPR monomer—a nucleotide should bulge from H93, at the same position that A2602 bulges from H93 in the ribosome (Figure 3c).

Additional base pairs formed among C-loop nucleotides were tolerated, as well as local changes within a helix.

### 4.3. Probability of Obtaining a Specific Sequence

The amount of random chains required in order to get at least one oligonucleotide with a predetermined sequence, with a probability larger than 99%, can be computed from the equation:

1 − (1 − P)^n^ > 0.99 (6)

where P is the probability of random occurrence of the sequence, and n is the number of oligonucleotides. Application of the polynomial approximation for infinitesimal P reduces this equation to P × n ≈ 1.

## Figures and Tables

**Table 1:** Results of the secondary structure predictions of the A-, P-monomeric sequences from the suggested proto-ribosomes from bacterial (*E. coli*, D50S, T50S), archaeal (H50S) and eukaryotic (*S. cer*) ribosomes. * indicate that the sequences of T50S and D50S are identical in that region, generating identical predictions.

Sequence	SymR		DPR		Ext-SymR	
	No. Solutions	Correct Shape of Best Solution	No. Solutions	Correct Shape of Best Solution	No. Solutions	Correct Shape of Best Solution
*E. coli*-A	3	√	2	√	3	√
*E. coli*-P	3	-	1	√	1	√
D50S, T50S-A	1 *	√ *	1 *	√ *	2 *	√ *
D50S-P	3 *	- *	1 *	√ *	1	√
T50S-P	3 *	- *	1 *	√ *	1	√
H50S-A	3	-	1	√	3	-
H50S-P	2	-	1	√	3	-
*S.cer*-A	2	-	1	-	2	-
*S.cer*-P	3	-	3	-	4	-
